# Efficacy and Tolerability of 6-Month Treatment with Tamsulosin Plus the Hexanic Extract of *Serenoa repens* versus Tamsulosin Plus 5-Alpha-Reductase Inhibitors for Moderate-to-Severe LUTS-BPH Patients: Results of a Paired Matched Clinical Study

**DOI:** 10.3390/jcm11133615

**Published:** 2022-06-22

**Authors:** Antonio Alcaraz, David Castro-Díaz, Mauro Gacci, Andrea Salonia, Vincenzo Ficarra, Joaquín Carballido-Rodríguez, Alfredo Rodríguez-Antolín, José Medina-Polo, Jesús M. Fernández-Gómez, José M. Cózar-Olmo, Santiago Búcar-Terrades, Noemí Pérez-León, Francisco J. Brenes-Bermúdez, José M. Molero-García, Antonio Fernández-Pro-Ledesma, Michael Herdman, Javier C. Angulo, José Manasanch, on behalf of the QUALIPROST Study Group

**Affiliations:** 1 Urology Department, Hospital Clínic, Universitat de Barcelona, IDIBAPS (Institut d’Investigacions Biomèdiques August Pi i Sunyer), 08036 Barcelona, Spain; aalcaraz@clinic.cat; 2 Urology Department, Hospital Universitario de Canarias, 38320 Tenerife, Spain; davidmanuelcastrodiaz@gmail.com; 3 Unit of Minimally Invasive and Robotic Urologic Surgery and Kidney Transplantation, Careggi University Hospital (AOUC), University of Florence, 50134 Florence, Italy; maurogacci@yahoo.it; 4 Department of Experimental and Clinical Medicine, University of Florence, 50134 Florence, Italy; 5 Division of Experimental Oncology/Unit of Urology, URI, IRCCS Ospedale San Raffaele, 20132 Milan, Italy; salonia.andrea@hsr.it; 6 Department of Human and Pediatric Pathology “Gaetano Barresi”, Urology Section, University of Messina, 98125 Messina, Italy; vficarra@unime.it; 7 Urology Department, Hospital Universitario Puerta de Hierro Majadahonda, 28222 Majadahonda, Spain; carballidojoaquin@gmail.com; 8 Urology Department, Research Institute i + 12, Hospital Universitario 12 de Octubre, 28041 Madrid, Spain; arantolin@yahoo.es (A.R.-A.); jose.medina@rocurologia.com (J.M.-P.); 9 Urology Unit, HM Hospital, 28050 Madrid, Spain; 10 ROC Clinic, 28010 Madrid, Spain; 11 Urology Department, Hospital Universitario Central de Asturias, 33011 Oviedo, Spain; jmfergomez@gmail.com; 12 Urology Department, Hospital Universitario Virgen de las Nieves, 18014 Granada, Spain; cozarjm@yahoo.es; 13 Urology Department, Hospital El Pilar, Quirónsalud, 08006 Barcelona, Spain; sbucarterrades@gmail.com; 14 Gran Sol Primary Care Center, 08914 Badalona, Spain; noemi612@gmail.com; 15 SEMERGEN Nefro-Urology Working Group, 08338 Premià de Dalt, Spain; fjbrenesb@gmail.com; 16 San Andrés Primary Care Center, 28021 Madrid, Spain; jmolerog@gmail.com; 17 Menasalbas Primary Care Center, 45128 Toledo, Spain; afernandezprol@semg.es; 18 Insight Consulting and Research, 08301 Mataró, Spain; michael.herdman@insightcr.com; 19 Saw Swee Hock School of Public Health, National University of Singapore, Singapore 117549, Singapore; 20 Clinical Department, Universidad Europea de Madrid, 28905 Getafe, Spain; javier.angulo@universidadeuropea.es; 21 Urology Department, Hospital Universitario de Getafe, 28905 Getafe, Spain; 22 SEMERGEN, 08329 Teià, Spain

**Keywords:** moderate-severe LUTS, BPH, combination therapy, tamsulosin, 5-alpha-reductase inhibitors, hexanic extract of *Serenoa repens*, quality of life, urinary symptoms, prostate

## Abstract

The objective of this subset analysis was to evaluate and compare the efficacy and tolerability of two combination treatments for men with moderate-to-severe lower urinary tract symptoms associated with benign prostatic hyperplasia (LUTS/BPH). Data were from a real-world, open-label, prospective, and multicenter study performed in outpatient urology clinics. Men with moderate-to-severe LUTS/BPH received 6-month treatment with tamsulosin (TAM) in combination with either the hexanic extract of *S. repens* (HESr) or a 5-alpha-reductase inhibitor (5ARI). Changes in urinary symptoms and quality of life were measured using the IPSS and BII questionnaires, respectively. Treatment tolerability was assessed by recording adverse effects (AEs). Patients in the two study groups were matched using iterative and propensity score matching approaches. After iterative matching, data were available from 136 patients (*n* = 68 treated with TAM + 5ARI, *n* = 68 with TAM + HESr). After 6 months of treatment, mean (SD) IPSS total score improved by 7.7 (6.3) and 6.7 (5.0) points in the TAM + 5ARI and TAM + HESr groups, respectively (*p* = 0.272); mean BII total scores improved by 3.1 (2.9) and 2.9 (2.4) points (*p* = 0.751), respectively. AEs were reported by 26.5% and 10.3% of patients in the same groups, mostly affecting sexual function (*p* < 0.027). When used in a real-world setting to treat patients with moderate-severe LUTS/BPH, 6-month treatment with TAM + HESr was as effective as TAM + 5ARI, but with better tolerability.

## 1. Introduction

Benign prostatic hyperplasia (BPH) is a frequent cause of lower urinary tract symptoms (LUTS) in adult men. LUTS/BPH can be highly bothersome, impairing the quality of life (QoL) of men with the condition, and that of their partners [1,2]. LUTS are strongly associated with ageing [1], and both the prevalence of LUTS and their associated costs are expected to rise in tandem with life expectancies [2,3]. 

Standard medical treatments for patients with uncomplicated LUTS/BPH include alpha-blockers (AB), 5-alpha-reductase inhibitors (5ARIs), phosphodiesterase type 5 inhibitors, antimuscarinics, and the beta-3 agonist mirabegron. The hexanic extract of *Serenoa repens* (HESr) is also recommended as a therapeutic option in the EAU Guidelines on non-neurogenic LUTS [1].

Medical treatments are usually prescribed as a monotherapy in patients with mild to moderate LUTS/BPH, though in patients showing insufficient improvement and/or more severe symptoms, treatments may be combined [1]. The most widely used combination therapy (CT) is an AB with a 5ARI [4], although it has been reported that adverse events are significantly more common with this combination than with ABs alone or 5ARIs alone [5].

While the HESr has proven as effective as ABs [6,7,8,9,10,11] and 5ARIs when used as a monotherapy over a 6-month period [7,8,12], it has a significantly superior tolerability profile [6,7,9,10,11,12], which may make it more attractive as a treatment option for LUTS/BPH. Of note, it is the sole extract of *S. repens* that the European Medicines Agency (EMA) considers as having sufficient evidence to support its use as a treatment for LUTS/BPH [13]. While it has been shown to be effective as a monotherapy, there has been limited research into its effectiveness when used in combination with other treatments for LUTS/BPH [14,15], especially in real-world settings [9,16]. The anti-inflammatory [17,18], 5-alpha reductase inhibitor [19,20], and antiproliferative [21] mechanisms of action reported for the HESr could lead to a synergistic mechanism of action when used in combination with an AB. As a persistent prostatic inflammatory state plays a role in the development and progression of LUTS/BPH [22], the anti-inflammatory effect of HESr might contribute to greater relief of LUTS/BPH symptoms than when using an AB alone [22].

The real-world QUALIPROST study [9] investigated the impact of LUTS/BPH and its treatment on symptoms and QoL in current clinical practice and found that the two most frequently used combination treatments were an AB, usually tamsulosin (TAM), together with a 5ARI or HESr. The aim of this sub-analysis of QUALIPROST data was to compare the efficacy and tolerability of 6-month treatment with TAM + 5ARI vs TAM + HESr.

## 2. Materials and Methods

### 2.1. Patients and Study Design

Data were from the QUALIPROST study [9] (ISRCTN11815680), a multicenter, non-interventional study performed between September 2009 and June 2011, to evaluate changes in symptoms and QoL in patients ≥40 years of age with bothersome moderate-to-severe LUTS/BPH managed in urology clinics. Patients were followed up for 6 months and were treated according to the usual clinical practice of the participating clinicians. The study complied with recommendations in the STROBE statement http://www.strobe-statement.org/ (accessed on 10 March 2022) and is described in detail in Alcaraz A et al. [9].

Data in the present sub-analysis were from patients with a baseline IPSS score of ≥12 points who received commercially available tamsulosin (Omnic^®^, Astellas Pharma Inc, Tokyo, Japan; Urolosin^®^, Boehringer Ingelheim, Ingelheim, Germany; or generics at a daily recommended dose [DRD] of 0.4 mg) in combination with either a 5ARI (dutasteride [Avidart^®^, GSK, London, England], at a DRD of 5 mg/day; or finasteride [Proscar^®^, MSD, Kenilworth, USA], at a DRD of 0.5 mg/day) or with the HESr (Permixon^®^, Pierre Fabre, Castres, France, at a DRD: 320 mg/day).

All procedures performed in the study were in accordance with the ethical standards of the institutional and/or national research committee and with the 1964 Helsinki declaration and its later amendments, or comparable ethical standards. Ethical approval was obtained from the Ethics Committee of the Puerta de Hierro Majadahonda University Hospital in Madrid, Spain. Individual informed consent was obtained from all participants in the study.

### 2.2. Procedures

Primary endpoints of the QUALIPROST study were change in LUTS, assessed using the IPSS, and change in QoL, evaluated using the BII. Improvements of >3.1 points on the IPSS and of >0.4 points on the BII were considered clinically relevant [23]. The BII and the IPSS were self-completed by patients at baseline and at the 6-month follow-up visit. Sociodemographic and clinical data, and adverse effects (AEs) potentially associated with treatment, were also recorded at baseline and at the 6-month follow-up.

### 2.3. Statistical Analysis 

As this was an observational study, in which patients were not randomized into the treatment groups, two matching approaches were used to ensure comparability. The first involved iterative matching of patients in the two groups, to ensure they were comparable at an aggregate level in terms of baseline IPSS (total, voiding and storage sub-scores, and item 8), BII, maximum urinary flow (Qmax), prostate volume, and prostate-specific antigen (PSA). Patients were removed from the TAM + 5ARI or TAM + HESr groups and *t*-tests were used to continually compare the two groups until no statistically significant differences (*p* > 0.10) were observed between them on any of the key baseline characteristics. To determine the success of the matching procedure, Student’s *t*-test was used to compare pre- and post-matching baseline scores on the IPSS and BII total scores, the IPSS voiding and storage sub-scores, and IPSS item 7 (nocturia) scores in the two groups. The second method was a propensity score matching procedure, in which each patient in the TAM + 5ARI group was paired with a patient from the TAM + HESr group with a similar likelihood of receiving TAM + 5ARI, estimated using a logistic regression model and including the same baseline characteristics used in the iterative procedure as independent variables.

Change over time within each group was assessed using paired *t*-tests; between-group differences in IPSS and BII change scores were assessed using *t*-tests for independent samples. Changes in items 1–7 on the IPSS, which assess symptom severity, were analyzed separately from item 8, which assesses QoL.

With a sample size of 68 patients in each treatment group, the study had a statistical power of 94.2% to detect a 6-month minus baseline mean IPSS difference of at least 3.1 units, assuming a type I error probability of 5% and a standard deviation of 5.1 units for the between-group difference in change scores.

Adverse effects were reported as absolute and relative frequencies, and comparisons between groups were assessed using a chi-square or exact Fisher test, as appropriate.

All analyses were performed separately for the final samples defined by the iterative and propensity score matching procedures. In the iteratively matched sample, the analysis was performed both for the sample as a whole and for a sub-group with more severe baseline symptoms (IPSS ≥ 20 points). 

Patients with any missing data on the IPSS and BII at any visit were excluded from the analysis, as were any patients who were lost to follow-up or that stopped or changed treatment. In all comparisons, results were considered statistically significant at *p* < 0.05. Statistical analyses were carried out using R 4.1.1 statistical software, R Core Team, R Foundation for Statistical Computing, Vienna, Austria; https://www.r-project.org (accessed on 7 March 2022).

## 3. Results

### 3.1. Iteratively Matched Sample

**Figure 1:**
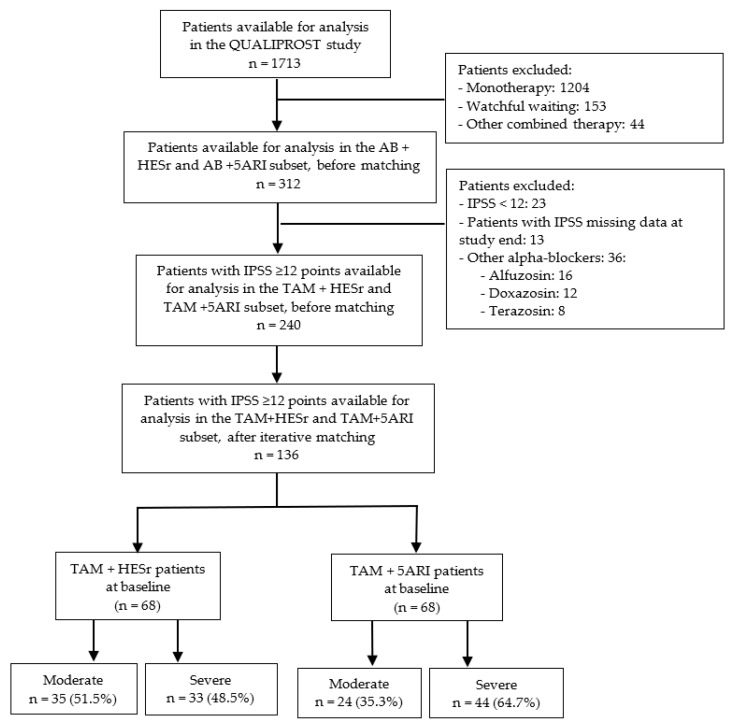
Study flow-chart (iterative matching sample). AB: alpha-blockers; TAM: tamsulosin; 5ARI; 5-alpha-reductase inhibitor; HESr: hexanic extract of *Serenoa repens*; IPSS: International Prostate Symptom Score.

A total of 136 patients were available for analysis (*n* = 68 receiving TAM + HESr, *n* = 68 receiving TAM + 5ARI) (Figure 1). Of patients receiving a 5ARI, 58 (85.3%) were treated with dutasteride and 10 (14.7%) with finasteride.

Table 1 shows the baseline characteristics for the two groups. There were no significant differences between the groups on any of the variables analyzed.

As shown in Table S1, 55.9% (*n* = 38) of patients had at least one concomitant disease in the TAM + HESr group compared to 45.6% (*n* = 31) of patients in the TAM + 5ARI group (*p* = 0.303). High blood pressure, dyslipidemia, and diabetes mellitus were the most frequent concomitant diseases and were reported by 27.9%, 29.4%, and 17.6% of patients, respectively, in the TAM + HESr arm, and by 19.1%, 17.6%, and 19.1% of patients in the TAM + 5ARI arm, with no statistically significant differences between the groups (*p* > 0.15).

**Figure 2:**
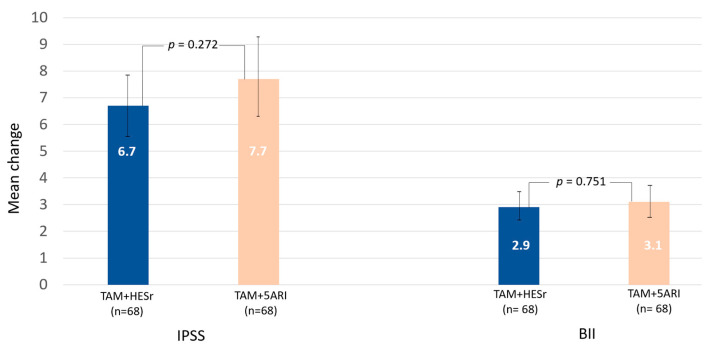
Mean IPSS and BII improvement (95% CI) in the two treatment groups after 6-month treatment (iterative matching sample). TAM: tamsulosin; 5ARI; 5-alpha-reductase inhibitor; HESr*:* hexanic extract of *Serenoa repens;* IPSS: International Prostate Symptom Score; BII: Benign Prostatic Hyperplasia Impact Index.

Figure 2 shows the mean (95% CI) IPSS and BII score changes for the two groups, after 6-month treatment. Mean (SD) IPSS score improved by 6.7 (5.0) points in the TAM + HESr group compared to 7.7 (6.3) for TAM + 5ARI. Mean (SD) BII improvement was 2.9 (2.4) and 3.1 (2.9) points, in the TAM + HESr and TAM + 5ARI groups, respectively. There were no significant differences between the groups for either endpoint.

Table 2 shows mean (SD) change scores for symptom and QoL measures by study group, after 6 months of treatment. Patients in both groups showed similar levels of improvement on all measures and no statistically significant differences for any of the outcomes.

As clinicians applied their usual criteria for requesting clinical tests, there were fewer Qmax, prostate volume, and PSA data available at follow-up than IPSS and BII data, and it was insufficient to allow for an appropriate analysis. The available data are nevertheless shown in Table S2 for information.

Change scores on symptom and QoL measures were also analyzed by treatment group for patients with more severe baseline symptoms (IPSS > 19). In this subgroup, patients improved by a mean (SD) of 9.2 (5.2) and 9.7 (6.2) points on IPSS (*p* = 0.678) and by a mean (SD) of 3.5 (2.3) and 3.8 (3.0) points on the BII (*p* = 0.577) in the TAM + HESr and TAM + 5ARI arms, respectively (Table S3).

In total, 82.64% of patients in the TAM + HESr group improved by at least 3 points on the IPSS compared to 80.9% in the TAM + 5ARI arm (*p* = 1.000). Likewise, 69.1% (TAM + HESr) and 66.2% (TAM + 5ARI) of men showed ≥25% improvement in IPSS score at the end of the study (*p* = 0.855 between groups). 

Table 3 shows that patients receiving TAM + 5ARI had a considerably higher rate (26.5%) of AEs than those receiving TAM + HESr (10.3%, *p* = 0.027). Erectile dysfunction, reduced libido, and anejaculation were the most frequent AEs in the TAM + 5ARI group.

### 3.2. Propensity Score Matched Sample

Results in the propensity score matched sample showed very similar results to those observed in the iteratively matched sample, although the patient sample available for analysis was limited (*n* = 25 in each group) (Figure S1 and Table S4). Improvements on all study outcomes were similar between the groups and in line with those seen in the iteratively matched sample. The efficacy results obtained with this analysis can be found in Supplementary Materials (Figure S2 and Table S5). 

## 4. Discussion

As far as we know, this is the first clinical study to compare changes in symptoms and QoL in patients with moderate-severe LUTS/BPH treated with either TAM + HESr or TAM + 5ARI for 6 months. Patients in the two groups showed similar levels of improvement in symptoms and QoL, whilst significantly more AEs were observed with TAM + 5ARI, particularly affecting sexual function. 

The improvement of 6.7 points on the IPSS observed here with TAM + HESr is similar to that observed in earlier studies which evaluated a combination of an AB and HESr. For example, Boeri et al. reported an improvement of 6.4 points on the IPSS in patients treated with silodosin + HESr [16] after a mean follow-up of 13.5 months, while Alcaraz A et al. [10] reported a mean improvement of 7.2 points for patients receiving TAM + HESr. The COMBAT study showed an improvement of 7.3 points on the IPSS at 4 years for patients treated with tamsulosin + dutasteride who completed the study [24], which is comparable to the 7.7 points of improvement seen in our study in the TAM + 5ARI arm. Similarly, the mean improvement in BII score in both groups in our study approximates the 2.2-point improvement observed in the COMBAT study with the same questionnaire [25].

The higher incidence of AEs in the TAM + 5ARI group observed in our study was most likely due to treatment with 5ARI [26]. The most frequent AEs were reduced libido, erectile dysfunction, and ejaculatory disturbances, which coincides with reports in other studies [5,27,28]. A high incidence of AEs related to sexual function, and their effect on QoL, could help explain the low adherence seen in the use of long-term CT. For instance, after 6 months of treatment, therapeutic persistence was reported at 45.5% in patients receiving an AB, 5ARI or CT, and 28.6% after 10 months, in a population-based cohort study [29]. Specifically for CT, only 8.2% of patients persisted with treatment after one year, and CT had the highest discontinuation rate in the first 2 years of treatment, in comparison with AB or 5ARI monotherapy (*p* < 0.0001), thereby highlighting the difficulty of maintaining long-term CT treatment. In another study in current clinical practice, it was reported that 20% of discontinuations by 12 months were associated with the presence of adverse events [30].

CT with TAM + 5ARIs is associated with decreased libido in 6% of patients receiving the treatment, erectile dysfunction in 9%, and ejaculatory dysfunction in 7% [24]. A meta-analysis that assessed the impact of medical treatments for LUTS/BPH on ejaculatory function, found that CT with ABs and 5-ARIs is associated with a three-fold increased risk of ejaculatory dysfunction compared with each treatment used individually [5]. The deterioration of sexual function associated with LUTS/BPH treatment significantly affects the quality of life of sexually active patients, who may reject this CT due to these types of AE, even though it is the most standard CT [31]. 

Additionally, it has been shown that men undergoing medical treatment for LUTS/BPH prefer treatment options with a low risk of adverse events; and that up to 93% prefer a treatment with no sexual side effects (erectile dysfunction, loss of libido, and ejaculatory dysfunction) [31]. Furthermore, a recent qualitative analysis showed that side effects affecting sexual function are of greater importance than non-sexual side effects for sexually active patients [32]. In that regard, HESr has been shown to improve symptoms and QoL in BPH patients, but with only limited side effects, which do not impact sexual function [1,7,10,11,33], and this could help to maintain adherence in patients worried about the effect of medical LUTS/BPH treatment on their sexual function. The HESr is recommended in the recent EAU Guidelines as a treatment “for men with LUTS who want to avoid any potential adverse events especially related to sexual function” [1]. Its use in combination with an AB does not appear to increase AEs affecting sexual function [10,14,16].

In relation to the preservation of sexual function in the treatment of LUTS/BPH, in recent years, new surgical treatments have been developed which reduce treatment-associated sexual dysfunction. Such developments clearly address patients’ wishes to maintain sexual function after LUTS/BPH surgery [34] and support findings that men with a higher level of sexual function were less likely to prefer surgery [31,35]. Among these surgical procedures, two promising techniques, prostatic urethral lift and ablation, appear to have no significant impact on sexual function [1].

Other reported risks with the use of 5ARIs include the possibility of developing a more serious form of prostate cancer [36,37], and increased risk of self-harm and depression, compared with men unexposed to 5ARI [38,39,40]. Moreover, there is some evidence that inhibition of 5-alpha reductases with finasteride or dutasteride may be associated with increased risk of incident idiopathic venous thromboembolism [41], acute coronary syndrome [42], insulin resistance, type 2 diabetes mellitus, dry eye disease [43], or osteoporosis, among other metabolic dysfunctions [44]. An effective treatment without those risks and AEs could potentially lead to greater therapeutic persistence, increased health benefits, and lower costs, although these aspects were not explicitly assessed in the present study. Of note, the latest EAU Guidelines on non-neurogenic LUTS clearly state that “all patients should be counselled about pharmacological treatment related adverse events in order to select the most appropriate treatment for each individual patient” [1].

A further relevant aspect is the use of CT with ABs and 5ARIs to reduce risk of progression. It has been shown that using AB and 5ARI in combination is superior to use of an AB alone for reducing the risk of acute urinary retention or need for surgery [24]. For that reason, CT may be recommended in men with moderate-to-severe LUTS and an increased risk of disease progression when long-term treatment is planned, even though it is associated with a higher rate of adverse events than an AB or a 5ARI alone [1]. It should be borne in mind, however, that approximately 83% of LUTS/BPH patients receiving placebo in RCTs did not show progression at 4 years [45]. Even in patients who were potentially at risk of progression, 78.5% of those treated solely with tamsulosin, which has not been shown to reduce the risk of AUR or surgery associated with LUTS/BPH, did not present clinical progression of BPH at 4 years [24]. Indeed, symptom worsening is by far the most frequently occurring progression event, and studies such as the Olmsted County Study have shown that serious outcomes, such as AUR and BPH-related surgery, are relatively infrequent in the LUTS/BPH population [46]. These findings suggest that some LUTS/BPH patients might receive TAM + 5ARI when it is not absolutely necessary and when an effective and better tolerated option is available. This is particularly true in benign disease, where treatment can be changed during follow-up if clinically advisable, based on disease evolution. Such an approach would align with studies showing that male LUTS patients consistently prefer less-invasive management options with a low risk of AEs, especially sexual function AEs [31]. Notably, EAU guidelines on LUTS diagnosis and treatment indicate that treatment for LUTS/BPH should be tailored to each patient’s symptomatology, comorbidities, and preferences, taking into account treatment tolerability [1].

A strength of the present study is the use of two different matching approaches to ensure that groups were comparable at baseline on relevant variables. The lack of statistically significant differences between study groups at baseline in this sub-analysis supports the robustness and reliability of the study results. Another strength is that data were collected under conditions of current clinical practice, which may make the findings more applicable in a real-world situation.

The present study also had some limitations. As it was an observational study, there was no randomization or blinding, though this was offset by using two different powerful patient matching techniques to obtain homogeneous treatment groups at baseline. The relatively short follow-up period of six months may also be considered a limitation, as the full effect of treatment might not be observed for the 5ARI and the HESr. However, significant IPSS, QoL, and Qmax improvement has been reported for dutasteride at 3 and 6 months [47,48]. With reference to CT, the CONDUCT study reported that, of the total improvement seen at 2 years on the IPSS and QoL measures with TAM + dutasteride, about 80% was observed after only 3 months of treatment [49]. Furthermore, the longer-term change in symptoms in the two CT groups investigated in the present study is likely to be similar, as a greater 5-alphareductase inhibition effect is also seen with the HESr if treatment is prolonged beyond 6 months. For example, Pytel et al. [50] found that IPSS improvement at 6-months was equivalent to 74.7% of the total improvement observed after 2 years of treatment.

The use of matching techniques meant that not all available patients from the QUALIPROST database were included for analysis, which could affect the external validity of the results. A descriptive analysis of the baseline characteristics of patients included and excluded after iterative matching showed, however, similar values in symptoms and QoL scores. The only noteworthy differences were for prostate volume, Qmax, and PSA, which reflected there being slightly more severe disease in the patients included in the analysis (see Table S6). This potentially reinforces the external validity of the results, as combination treatment is recommended for moderate-to-severe LUTS patients who are at risk of disease progression [1]. Furthermore, results from the present analysis in terms of improvements in symptoms and QoL align quite closely with the results from other clinical studies and observations in clinical practice [10,14,16,24].

Two final limitations are the relatively low number of patients included in the propensity score matching procedure and the limited numbers of Qmax, prostate volume, and PSA evaluations carried out at follow-up. The low numbers in the propensity score matching procedure confer a low level of evidence for the results, and they are presented solely as support for those obtained with the iterative matching approach. The lower rates of Qmax, prostate volume, and PSA evaluations carried out at follow-up appear to simply reflect real-word practice. For example, a recently published article evaluating adherence to American Urological Association guidelines for evaluation and testing of LUTS/BPH, reported that the PSA test, which was the second most common test, was performed in as few as 15–34% of patients with LUTS/BPH [51]. 

## 5. Conclusions

In patients with moderate-severe LUTS/BPH managed in a real-life setting, 6-month treatment with TAM + HESr and TAM + 5ARI showed similar levels of improvement in symptoms and QoL, though with considerably fewer adverse effects in the TAM-HESr group, in particular regarding the preservation of libido. TAM + HESr therefore appears to be a valid therapeutic option for use in these patients over a 6-month period, though these results should be confirmed in an appropriately designed randomized controlled trial.

Based on the results of this sub-analysis, the TAM + HESr combination could be useful in patients with moderate-to-severe LUTS/BPH who are not at risk of progression but who might obtain additional symptom relief from a CT. Likewise, the TAM-HESr combination could be used in patients who are at risk of progression but who do not wish to follow the standard treatment due to its potential adverse effects on sexual function.

## Figures and Tables

**Table 1:** Patient baseline characteristics by treatment group (iterative matching sample).

	TAM + HESr		TAM + 5ARI		*p* Value
	*n*	Mean (SD)	*n*	Mean (SD)	
Age (years)	58	67.9 (7.9)	67	68.3 (7.3)	0.744
BMI (Kg/m^2^)	57	27.5 (3.1)	66	26.8 (2.8)	0.181
IPSS total (points)	68	20.2 (5.0)	68	21.5 (5.2)	0.137
*IPSS voiding sub-score*	68	11.6 (3.1)	68	12.5 (3.5)	0.116
*IPSS storage sub-score*	68	8.5 (2.3)	68	8.9 (2.3)	0.302
IPSS 8 (QoL)	68	4.1 (0.9)	68	4.1 (1.1)	0.804
Nocturia	68	2.9 (1.0)	68	3.0 (1.0)	0.792
BII (points)	68	8.0 (2.2)	68	8.4 (2.5)	0.338
Prostate volume (cm^3^)	54	68.6 (12.9)	66	73.2 (21.9)	0.148
Qmax (mL/s)	30	12.4 (3.2)	36	11.2 (5.0)	0.237
PSA (ng/mL)	63	2.9 (1.5)	62	3.2 (1.3)	0.244

TAM: tamsulosin; 5ARI: 5-alpha-reductase inhibitor; HESr: hexanic extract of *Serenoa repens*; SD: standard deviation; BMI: body mass index; IPSS: International Prostate Symptom Score; BII: Benign Prostatic Hyperplasia Impact Index; QoL: quality of life; Qmax: maximum urinary flow rate; PSA: prostate-specific antigen.

**Table 2:** Changes from baseline to 6-months in symptoms and quality of life by treatment group (iterative matching sample).

	TAM + HESr		TAM + 5ARI		*p* Value
	*n*	Mean (SD)	*n*	Mean (SD)	
IPSS total (points)	68	6.7 (5.0)	68	7.7 (6.3)	0.272
*IPSS voiding sub-score*	68	3.8 (3.3)	68	4.8 (4.0)	0.143
*IPSS storage sub-score*	68	2.8 (2.2)	68	3.0 (2.6)	0.726
IPSS 8 (QoL)	68	1.7 (1.2)	68	1.7 (1.3)	0.906
Nocturia	68	1.0 (1.0)	68	1.0 (1.1)	0.689
BII (points)	68	2.9 (2.4)	68	3.1 (2.9)	0.751

TAM: tamsulosin; 5ARI: 5-alpha-reductase inhibitor; HESr: hexanic extract of *Serenoa repens*; SD: standard deviation; IPSS: International Prostate Symptom Score; BII: Benign Prostatic Hyperplasia Impact Index; QoL: quality of life.

**Table 3:** Reported adverse effects for the study sample overall and by treatment group.

	TAM + HESr	TAM + 5ARI	*p* Value
	*n* = 68	*n* = 68	
Any adverse effect	7 (10.3%)	18 (26.5%)	0.027 *
Reduced libido	0 (0.00%)	8 (11.8%)	0.006 *
Erectile dysfunction	3 (4.41%)	7 (10.3%)	0.324
Anejaculation	2 (2.94%)	7 (10.3%)	0.165
Reduced ejaculatory volume	0 (0.00%)	4 (5.88%)	0.119
Orthostatic hypotension	2 (2.94%)	2 (2.94%)	1.000
Hypotension	1 (1.47%)	1 (1.47%)	1.000
Dizziness	1 (1.47%)	2 (2.94%)	1.000
Breast pain on palpation	0 (0.00%)	1 (1.47%)	1.000

* Statistically significant. TAM: tamsulosin; 5ARI; 5-alpha-reductase inhibitor; HESr: hexanic extract of *Serenoa repens.*

## Data Availability

The data underlying this article will be shared on reasonable request to the corresponding author.

## Supplementary Materials

The following supporting information can be downloaded at: https://www.mdpi.com/article/10.3390/jcm11133615/s1, Figure S1: Study flow-chart for the propensity score matched sample; Figure S2: Mean (95% CI) improvement in IPSS and BII in the two treatment groups (propensity score matching sample); Table S1: Concomitant diseases at baseline by treatment group, n (%), in the iterative matched sample; Table S2: Improvement from baseline to 6-month follow-up in PSA, Qmax and prostate volume for the study groups (iterative matching sample); Table S3: Improvements from baseline to 6-month follow-up in symptoms and quality of life by treatment group, in patients with severe (IPSS > 19) baseline symptoms (iterative matching sample); Table S4: Patient baseline characteristics by treatment group in the propensity score matched sample; Table S5: Improvements in symptoms, quality of life, and nocturia from baseline to 6-month follow-up by treatment group (propensity score matching sample); Table S6. Baseline characteristics for patients included and excluded from analysis after the iterative matching procedure.

## Appendix A

The following investigators participated in the QUALIPROST Study Group:

A Coruña: Dr. L. Busto Castañón; Dr. J.E. Duarte Novo; Dr. M. Montes Couceiro; Dr. A. Rodríguez Alonso; Alicante: Dr. J.A. Canovas Ivorra; Dr. C. López López; Dr. L. Prieto Chaparro; Almería: Dr. F. Gómez Berjón; Dr. J. Hortelano Parras; Asturias: Dr. A.M. Huescar; Dr. P.L. Muntañola Armora; Badajoz: Dr. E. Godoy Rubio; Dr. J. Mariño del Real; Barcelona: Dr. J. Armona Mani; Dr. F.J. Blasco Casares; Dr. Ll. Cortadellas Angel; Dr. J. Fernández Zuazu; Dr. J.M. Malet Carreras; Dr. S. Mando Dakkar; Dr. J.M. Prats Puig; Dr. M. Puyol Pallás; Dr. J.A. Romero Martín; Dr. J. Sáenz de Cabezón Martí; Dr. J. Sánchez Macías; Dr. C. Vargas Blasco; Dr. J.M. Vayreda Martija; Bilbao: Dr. J.A. Gallego Sánchez; Dr. N. Prieto Ugidos; Cádiz: Dr. J.M. Arroyo Maestre; Dr. S. Garrido Insua; Dr. A. Gutiérrez de Pablo; Dr. M. Marmol Ruíz; Dr. F. Reyes Martínez; Castellón: Dr. J. Beltrán Persiva; Dr. L. Monzonis Rey; Dr. J. Palacios Castañeda; Ciudad Real: Dr. N. Jiménez López-Lucendo; Dr. D. Rodríguez Leal; Córdoba: Dr. J.C. Regueiro López; Granada: Dr. M. Cabezas Zamora; Guipúzcoa: Dr. G. Garmendia Olaizola; Dr. J.A. Rodríguez Andrés; Jaén: Dr. J. Jiménez Verdejo; Dr. E.J. Zarate Rodríguez; León: Dr. S.C. Gómez Cisneros; Dr. M. Lozano Rebollo; Lleida: Dr. J. Cortada Robert; Dr. L.M. Flavian Domenech; Logroño: Dr. A. Fernández Fernández; Lugo: Dr. F.J. Neira Pampin; Madrid: Dr. A. Abdallah Merhi; Dr. F. Arias Funes; Dr. I.T. Castillón Vela; Dr. J.M. Duarte Ojeda; Dr. M.J. García-Matres Cortés; Dr. J.F. Hermina Gutierrez; Dr. J.J. López-Tello García; Dr. C. Martín García; Dr. B.M.El-Awadeh; Dr. J.D. Rendón Sánchez; Dr. R. Rodríguez-Patron Rodríguez; Dr. J.C. Ruíz de la Roja; Dr. J. Vallejo Herrador; Dr. D. Vázquez Alba; Málaga: Dr. A. Bonilla Maldonado; Dr. J.M. Fernández Montero; Dr. A. Galacho Bech; Dr. C. Marchal Escalona; Dr. P. Rodero García; Dr. G. Sanz; Dr. J.D. Vázquez Cervilla; Murcia: Dr. G. Server Pastor; Pontevedra: Dr. D. Jamardo González; Dr. A. Selas Pérez; Salamanca: Dr. F. Díaz Alférez; Dr. M.F. Lorenzo Gómez; Santander: Dr. J.A. Portillo Martín; Sevilla: Dr. F.J. Giráldez Puig; Dr. M.A. Gutiérrez González; Dr. J.I. Huesa Martínez; Dr. Y. Ismail Tomaizeh; Dr. J. Leal Arenas; Dr. J. Martín Calero; Dr. J.L. Moyano Calvo; Dr. A. Ortiz Gámiz; Dr. J.M. Poyato Galán; Dr. J.A. Valero Puerta; Dr. E. Vilches Cocovi; Tarragona: Dr. P.L. Álvarez de la Red; Dr. J. Benagues Pamies; Dr. M. Prados Saavedra; Tenerife: Dr. T. Concepción Masip; Dr. C. Gómez de Segura Melcon; Dr. E. González de Chaves; Dr. J. Postius Robert; Toledo: Dr. F. Álvarez Fernández; Dr. A. Iglesias Justo; Dr. A. Melchor Galán; Valencia: Dr. J.E. Blasco Alfonso; Dr. M.A. Bonillo García; Dr. A. Collado Serra; Dr. J.A. García Cebrián; Dr. L.García Reboll; Dr. Y. Pallás Costa; Dr. M.J. Sánchez Sanchiz; Dr. J. Santamaría Meseguer; Zaragoza: Dr. A. García de Jalón Martínez; Dr. J.M. Gil Fabra; Dr. F. Monzón Alebesque; Dr. A. Ucar Terren.
