# Supplementary material for: Efficacy and Tolerability of 6-Month Treatment with Tamsulosin Plus the Hexanic Extract of Serenoa repens versus Tamsulosin Plus 5-Alpha-Reductase Inhibitors for Moderate-to-Severe LUTS-BPH Patients: Results of a Paired Matched Clinical Study
Source: J Clin Med. 2022 Jun 22;11(13):3615. doi: 10.3390/jcm11133615 (PMC9267652; doi:10.3390/jcm11133615)
Supplement: Supplementary file 1 [file jcm-11-03615-s001.zip › jcm-1736154-supplementary.pdf]

Supplementary Materials

Table S1. Concomitant diseases at baseline by treatment group, n (%), in the iterative matched sample.

	**TAM+HESr** (n = 68)	**TAM+5ARI** (n = 68)	***p* Value**
Patients with any concomitant illness, n (%)	38 (55.9)	31 (45.6)	0.303
High blood pressure	19 (27.9)	13 (19.1)	0.312
Dyslipidemia	20 (29.4)	12 (17.6)	0.157
Diabetes mellitus	12 (17.6)	13 (19.1)	1.000
Other	3 (4.41)	0 (0.0)	0.244

TAM: tamsulosin; 5ARI; 5-alpha-reductase inhibitor; HESr: hexanic extract of *Serenoa repens*

Table S2. Improvement from baseline to 6-month follow-up in PSA, Qmax and prostate volume for the study groups (iterative matching sample).

	**TAM+HESr**		**TAM+5ARI**		***p* Value**
	**n***	**Mean (SD)**	**n***	**Mean (SD)**	
Qmax (ml/sec)	9	1.6 (1.7)	21	2.2 (5.6)	0.670
Total PSA (ng/ml)	28	-0.7 (1.4)	28	-0.9 (1.1)	0.644
Prostate volume (cm^3)	12	12.3 (13.7)	30	7.2 (10.2)	0.261

TAM: tamsulosin; 5ARI; 5-alpha-reductase inhibitor; HESr: hexanic extract of *Serenoa repens;* Qmax: maximum urinary flow rate; PSA: prostate-specific antigen.

* Number of patients vary according to the test and the personal clinical practice of the investigators.

Table S3. Improvements from baseline to 6-month follow-up in symptoms and quality of life by treatment group, in patients with severe (IPSS > 19) baseline symptoms (iterative matching sample).

	TAM+HESr		TAM+5ARI		*p* Value
	**n**	**Mean (SD)**	**n**	**Mean (SD)**	
IPSS total (points)	33	9.2 (5.2)	44	9.7 (6.2)	0.678
IPSS voiding sub-score	33	5.5 (3.2)	44	6.0 (4.0)	0.499
IPSS storage sub-score	33	3.7 (2.3)	44	3.7 (2.6)	0.979
IPSS 8 (QoL)	33	2.0 (1.1)	44	1.9 (1.3)	0.735
Nocturia	33	1.4 (1.1)	44	1.2 (1.1)	0.589
BII (points)	33	3.5 (2.3)	44	3.8 (3.0)	0.577

TAM: tamsulosin; 5ARI; 5-alpha-reductase inhibitor; HESr: hexanic extract of *Serenoa repens*; IPSS: International Prostate Symptom Score; BII: Benign Prostatic Hyperplasia Impact Index; QoL: Quality of Life.

Table S4. Patient baseline characteristics by treatment group in the propensity score matched sample.

	TAM+HESr		TAM+5ARI		*p* Value
	**n**	**Mean (SD)**	**n**	**Mean (SD)**	
Age, mean (SD) years	20	63.5 (7.5)	25	65.4 (6.1)	0.354
BMI (Kg/m^2), mean (SD)	19	26.7 (2.8)	24	26.9 (2.5)	0.842
IPSS, mean (SD)	25	22.0 (5.8)	25	22.6 (4.9)	0.674
IPSS voiding subscore	25	13.0 (3.3)	25	12.9 (3.2)	0.931
IPSS storage subscore	25	9.0 (2.8)	25	9.7 (2.3)	0.328
IPSS item 8 (QoL)	25	4.1 (1)	25	4.1 (1.2)	1.000
Nocturia	25	2.8 (1.1)	25	3.1 (1.0)	0.346
BII, mean (SD)	25	8.5 (1.9)	25	8.6 (2.4)	0.897
Prostate volume (cm^3)	25	66.2 (21.1)	25	66.8 (20.9)	0.920
Qmax (ml/s)	25	11.4 (3.2)	25	11.6 (3.4)	0.832
PSA (ng/ml)	25	2.7 (1.5)	25	2.7 (1.1)	0.933

TAM: tamsulosin; 5ARI; 5-alpha-reductase inhibitor; HESr: hexanic extract of *Serenoa repens.* BMI: body mass index; IPSS: International Prostate Symptom Score; BII: Benign Prostatic Hyperplasia Impact Index; QoL: quality of life; Qmax: maximum urinary flow rate; PSA: prostate-specific antigen.

Table S5. Improvements in symptoms, quality of life, and nocturia from baseline to 6-month follow-up by treatment group (propensity score matching sample).

	TAM+HESr		TAM+5ARI		*p*
	n	Mean (SD)	n	Mean	Value
IPSS total	25	7.9 (5.7)	25	9.6 (6.7)	0.336
IPSS voiding sub-score	25	4.8 (3.6)	25	5.7 (4.5)	0.451
IPSS storage sub-score	25	3.0 (2.5)	25	3.9 (2.6)	0.257
IPSS item 8	25	1.6 (1.0)	25	1.9 (1.4)	0.499
Nocturia	25	0.8 (0.9)	25	1.4 (1.1)	0.048
BII total	25	3.0 (2.6)	25	4.5 (2.8)	0.057

TAM: tamsulosin; 5ARI; 5-alpha-reductase inhibitor; HESr: hexanic extract of *Serenoa repens;* IPSS: International Prostate Symptom Score; BII: Benign Prostatic Hyperplasia Impact Index; Qmax: maximum urinary flow rate; PSA: prostate-specific antigen.

Table S6. Baseline characteristics for patients included and excluded from analysis after the iterative matching procedure.

	Included		Excluded	
	n	Mean (SD)	n	Mean (SD)
Age (years)	125	68.1 (7.6)	93	62.9 (7.4)
BMI (Kg/m^2)	123	27.1 (3.0)	92	26.8 (2.7)
IPSS total (points)	136	20.8 (5.1)	104	19.3 (5.1)
IPSS voiding sub-score	136	12.1 (3.3)	104	10.9 (3.4)
IPSS storage sub-score	136	8.7 (2.3)	104	8.4 (2.3)
IPSS 8 (QoL)	136	4.1 (1.0)	104	3.9 (1.0)
Nocturia	136	2.9 (1.0)	104	2.6 (0.9)
BII (points)	136	8.2 (2.3)	104	8.5 (2.1)
Prostate volume (cm^3)	120	71.1 (18.5)	103	48.6 (16.3)
Qmax (ml/s)	66	11.7 (4.3)	76	13.2 (3.0)
PSA (ng/ml)	125	3.0 (1.4)	101	2.0 (1.2)

BMI: body mass index; IPSS: International Prostate Symptom Score; BII: Benign Prostatic Hyperplasia Impact Index; QoL: Quality of Life; Qmax: Maximum Urinary Flow Rate; PSA: Prostate-Specific Antigen.

Supplementary figure

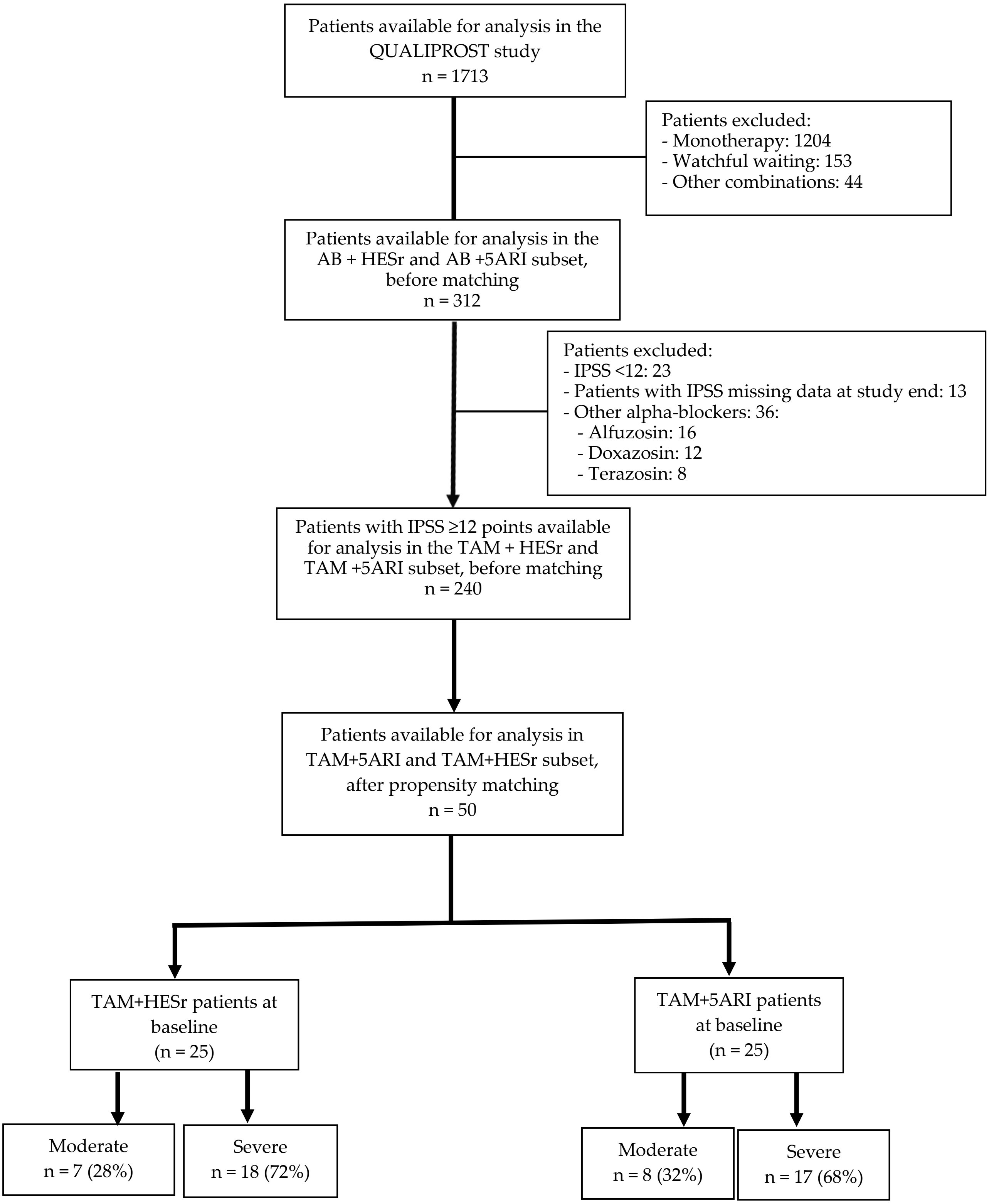

Figure S1. Study flow-chart for the propensity score matched sample.
TAM: tamsulosin; 5ARI; 5-alpha-reductase inhibitor; HESr: hexanic extract of *Serenoa repens.*

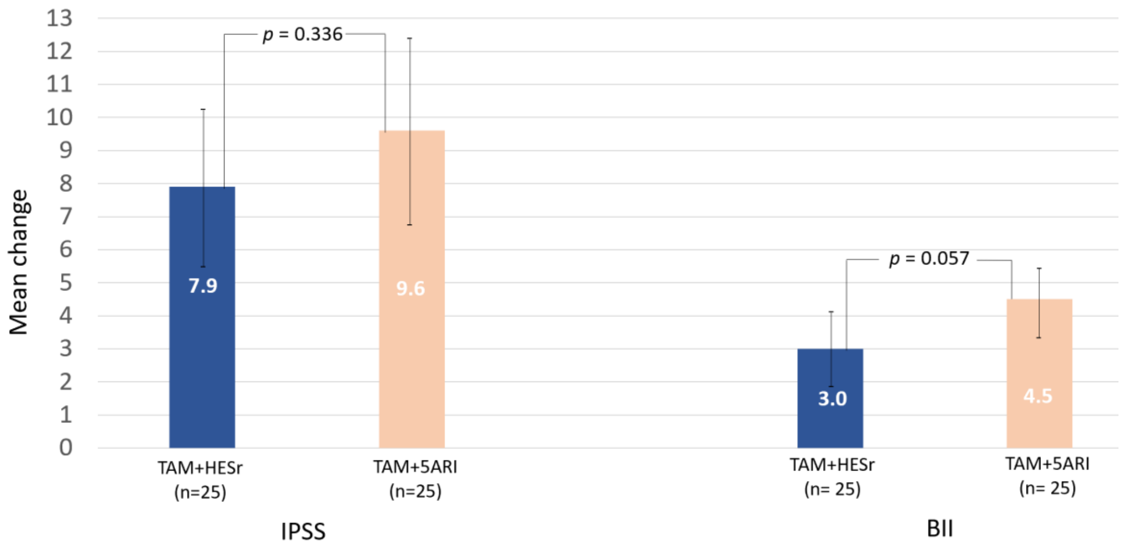

Figure S2. Mean (95% CI) improvements on the IPSS and BII in the two treatment groups (propensity score matching sample).

TAM: tamsulosin; 5ARI; 5-alpha-reductase inhibitor; HESr*:* hexanic extract of *Serenoa repens;* IPSS: International Prostate Symptom Score; BII: Benign Prostatic Hyperplasia Impact Index.
